# Genome survey and microsatellite motif identification of *Pogonophryne albipinna*

**DOI:** 10.1042/BSR20210824

**Published:** 2021-07-20

**Authors:** Euna Jo, Yll Hwan Cho, Seung Jae Lee, Eunkyung Choi, Jinmu Kim, Jeong-Hoon Kim, Young Min Chi, Hyun Park

**Affiliations:** 1 Department of Biotechnology, College of Life Sciences and Biotechnology, Korea University, Seoul 02841, Korea; 2 Division of Life Sciences, Korea Polar Research Institute (KOPRI), Yeonsu-gu, Incheon 21990, Korea

**Keywords:** GC content, genome assembly, genome size, microsatellite, Pogonophryne albipinna

## Abstract

The genus *Pogonophryne* is a speciose group that includes 28 species inhabiting the coastal or deep waters of the Antarctic Southern Ocean. The genus has been divided into five species groups, among which the *P. albipinna* group is the most deep-living group and is characterized by a lack of spots on the top of the head. Here, we carried out genome survey sequencing of *P. albipinna* using the Illumina HiSeq platform to estimate the genomic characteristics and identify genome-wide microsatellite motifs. The genome size was predicted to be ∼883.8 Mb by K-mer analysis (K = 25), and the heterozygosity and repeat ratio were 0.289 and 39.03%, respectively. The genome sequences were assembled into 571624 contigs, covering a total length of ∼819.3 Mb with an N50 of 2867 bp. A total of 2217422 simple sequence repeat (SSR) motifs were identified from the assembly data, and the number of repeats decreased as the length and number of repeats increased. These data will provide a useful foundation for the development of new molecular markers for the *P. albipinna* group as well as for further whole-genome sequencing of *P. albipinna*.

## Introduction

The genus *Pogonophryne* Regan, 1914 is the most species-rich group among the perciform suborder Notothenioidei, with 28 species reported to date [1,2]. They inhabit coastal or deep waters of the Southern Ocean off Antarctica [2]. Recently, several species have been newly discovered during longlining of the Antarctic toothfish, *Dissostichus mawsoni* [1–7], but their morphological and molecular identification is still complicated.

Taxonomically, the genus *Pogonophryne* is one of the complex taxa distinguished from other taxa by slight meristic differences, and their key diagnostic character, namely the mental barbell, is highly variable in some species [6,8]. It is difficult to compare the morphology of the species from this genus because many of them were described based on only a few specimens from a single sampling site [9,10]. Accordingly, taxonomists have divided the genus *Pogonophryne* into five species groups: *P. mentella, P. scotti, P. barsukovi, P. marmorata*, and *P. albipinna* groups [5,11].

Phylogenetic studies have been carried out on these groups using several mitochondrial and nuclear markers, and the monophyly of these five species groups was supported by mitochondrial NADH dehydrogenase subunit 2 (ND2) and cytochrome *c* oxidase I (COI) gene markers [5,10]. However, molecular identification at the species level showed poor resolution due to low genetic variations related to a very recent divergence of the genus *Pogonophryne*, as is the case with other species in the family Artedidraconidae [10,12–14]. Therefore, it is necessary to develop markers with improved discriminatory ability for genome-wide analyses, such as microsatellite and single nucleotide polymorphism (SNP) markers. In particular, microsatellites, also termed simple sequence repeats (SSRs), have already been validated for their effectiveness in fish species delimitation [15].

The molecular data on *Pogonophryne*, mostly mitochondrial ND2 and COI, are available from the NCBI GenBank database [2,5] for less than half of the species (13 out of 28). Among these species, *P. albipinna* has been reported recently with its complete mitochondrial genome sequence [16], and this is the first genome survey study of *Pogonophryne. Pogonophryne albipinna*, also known as white-fin plunderfish, belongs to the *P. albipinna* group, which is the most deep-living group of the genus and is mainly characterized by an absence of dark spots on the top of the head [1,5,11].

In the present study, based on next-generation sequencing (NGS), we estimated the genomic characteristics of *P. albipinna* and identified genome-wide SSR motifs. The present study can be used as a basis for further whole-genome sequencing of *P. albipinna* and the development of new molecular markers for distinguishing between *Pogonophryne* species.

## Materials and methods

### Sample preparation and genome survey sequencing

Sample of *P. albipinna* was collected from the Ross Sea (77°05′S, 170°30′E on CCAMLR Subarea 88.1), Antarctica and frozen while being transferred to the laboratory. The frozen sample was dissected to obtain muscle tissue samples, which were used to extract genomic DNA following the traditional phenol-chloroform method. DNA quantity and quality were checked using a Qubit fluorometer (Invitrogen, Life Technologies, CA, U.S.A.) and a fragment analyzer (Agilent Technologies, CA, U.S.A.). Species were identified by morphology as well as using mitochondrial COI markers [17]. The DNA was randomly fragmented into 350-bp fragments using a Covaris M220 focused-ultrasonicator (Covaris, MA, U.S.A.). A paired-end DNA library was prepared and sequenced on the Illumina HiSeq 2000 platform according to the manufacturer’s protocol.

### Data analysis

The quality values of Q20 (percentage of bases whose base call accuracy exceeds 99%) and Q30 (percentage of bases whose base call accuracy exceeds 99.9%) and the GC content were evaluated from the primary Illumina paired-end data. K-mer analysis was conducted using Jellyfish 2.1.4 [18] with K-values of 17, 19, and 25. In order to estimate the genome size, heterozygosity rate and repeat content, we used GenomeScope [19] in R version 3.4.4 [20] based on the K-mer distribution (K = 25), which selected the one that the GenomeScope model showed the best match to the observed K-mer frequencies. The *de novo* draft genome was assembled using Maryland Super-Read Celera Assembler (MaSuRCA) version 3.3.4 [21], and contig-level assembly statistics were then calculated using the assemblathon_stats.pl script (available at: https://github.com/ucdavis-bioinformatics/assemblathon2-analysis/blob/master/assemblathon_stats.pl; accessed on 1 January 2021) [22]. Genome-wide identification of di- to hexanucleotide microsatellite motifs with minimum five repetitions, and primer design were performed using the pipelines of QDD version 3.1.2 [23]. Microsatellites were extracted with 200-bp flanking regions on both sides and sequences shorter than 80 were eliminated. Three QDD steps were proceeded with default parameters, and -contig 1 (step 1), -make_cons 0 (step 2) and -contig 1 (step 3) options were added. Primer pairs were selected by Primer3 software [24] to meet the following criteria: the expected PCR product size of 100–150 bp, the primer melting temperature (Tm) of 59–60°C, and the primer length of 20–25 bases.

## Results and discussion

### Genome size estimation and sequence assembly

**Figure 1:**
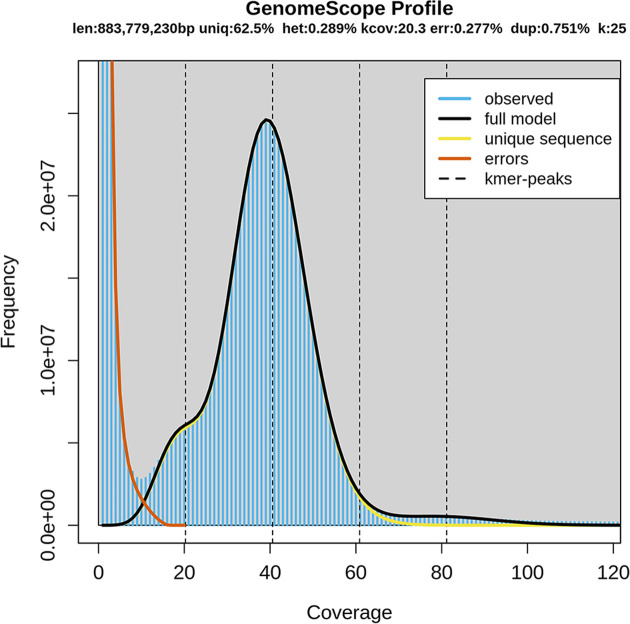
K-mer (K = 25) distribution of *P. albipinna* genome Blue bars represent the observed K-mer distribution; black line represents the modeled distribution without the error K-mers (indicated by the red line), up to a maximum K-mer coverage specified in the model (indicated by the yellow line). Len, estimated total genome length; Uniq, unique portion of the genome (not repetitive); Het, heterozygosity rate; Kcov, mean K-mer coverage for heterozygous bases; Err, error rate; Dup, duplication rate.

The genome survey sequencing of *P. albipinna* yielded a total of ∼57.1 Gb of raw reads through the Illumina paired-end library (Table 1). The Q20 and Q30 values of the raw reads were 96.6 and 91.8%, respectively (Table 1), indicating the high quality of this genome sequencing data [25]. In addition, the GC content of the raw reads was 41.7% (Table 1). The Illumina paired-end data were then used to predict the genomic characteristics of *P. albipinna* by K-mer analysis. Based on the 25-mer frequency distribution, the genome size was estimated to be 883.8 Mb, and the heterozygous and repetitive sequence rates were 0.289 and 0.751%, respectively (Table 2, and Figure 1).

**Table 1:** Statistics of the genome survey sequencing data of *P. albipinna*

Raw data (bp)	Total reads	Q20 (%)	Q30 (%)	GC content (%)
57104280342	378174042	96.6	91.8	41.7

**Table 2:** Genome estimation based on K-mer analysis of *P. albipinna*

K-mer	Genome size (bp)	Heterozygosity (%)	Duplication ratio (%)
17	829857227	0.275	0.795
19	843219952	0.294	0.758
25	883779230	0.289	0.751

In earlier studies, the nuclear DNA content of *P. scotti* was measured to be 4.05 pg/diploid cell using the Feulgen staining method [26]. When this measurement is converted into the haploid genome size, it shows that the nuclear DNA content of this species is 1.98 Gb, which is more than twice as high as our estimate. Meanwhile, other research on notothenioid genome size by flow cytometry showed that their genome size was 0.78–1.43 Gb [27], and more recent studies based on NGS data indicated a genome size of 0.64–1.06 Gb [28–32]. These size ranges are comparable with those indicated by our results, suggesting that further studies are needed to acquire more accurate knowledge of *P. albipinna* genome size.

Furthermore, the Illumina paired-end sequences of *P. albipinna* were assembled into contigs using MaSuRCA. We obtained 571624 contigs with a total length of 819289238 bp. The maximum and N50 contig lengths were 51460 and 2867 bp, respectively, with a GC content of 41.02% (Table 3). These results of genome survey sequencing provide useful preliminary data for further whole-genome studies to achieve more thorough assembly and chromosomal-level scaffolding using novel state-of-the-art genetic techniques.

**Table 3:** Statistics of the assembled genome sequences of *P. albipinna*

	Total length (bp)	Total number	Max length (bp)	N50 length (bp)	GC content (%)
**Contig**	819289238	571624	51460	2867	41.02

### Microsatellite motif identification

**Figure 2:**
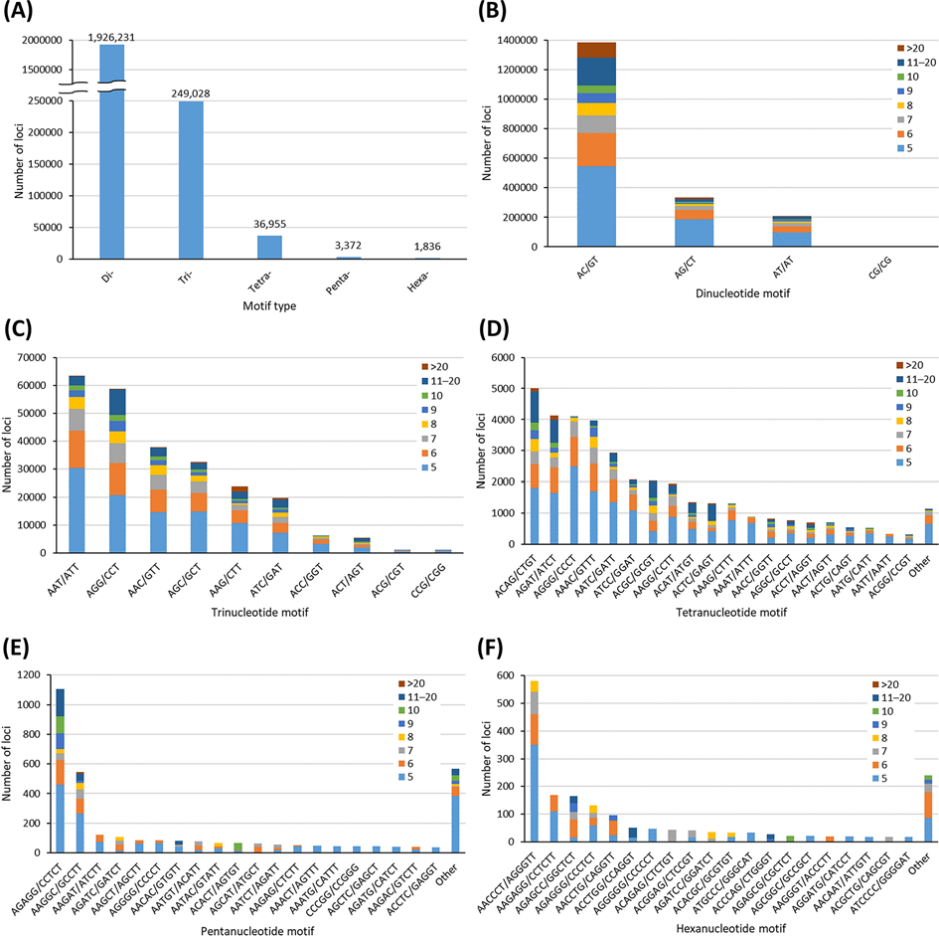
Type and frequency of microsatellite motifs in *P. albipinna* genome (**A**) Frequency of different microsatellite motif types. (**B**) Frequency of different dinucleotide microsatellite motifs. (**C**) Frequency of different trinucleotide microsatellite motifs. (**D**) Frequency of different tetranucleotide microsatellite motifs. (**E**) Frequency of different pentanucleotide microsatellite motifs. (**F**) Frequency of different hexanucleotide microsatellite motifs.

A total of 2217422 microsatellite motifs were identified from the genome assembly of *P. albipinna*. Among them, dinucleotide motifs were the most prevalent (1926231; 86.87%), followed by trinucleotides (249028; 11.23%), tetranucleotides (36955; 1.67%), pentanucleotides (3372; 0.15%), and hexanucleotides (1836; 0.08%) (Table 4 and Figure 2A). The tendency of the motif frequency in the studied species was similar to that in other fish species, with the dinucleotide motif being predominant [33,34]. In the dinucleotides, the most frequent motif was AC/GT (71.84%), followed by AG/CT (17.29%), AT/AT (10.82%), and CG/CG (0.05%) (Figure 2B). In the trinucleotides, the most frequent motif was AAT/ATT (25.43%), followed by AGG/CCT (23.57%), and AAC/GTT (15.09%) (Figure 2C). The most abundant motifs in the tetra-, penta-, and hexanucleotides were ACAG/CTGT (13.53%), AGAGG/CCTCT (32.80%), and AACCCT/AGGGTT (31.92%), respectively (Figure 2D–F). Information on 99 pairs of microsatellite marker is presented in Supplementary Table S1. To ensure the usability of the microsatellite markers, subsequent validation studies are required. Moreover, if these markers are applied for studying the *P. albipinna* group, more meaningful results could be obtained and interspecific variation could be explained better than when using conventional mitochondrial markers.

**Table 4:** Statistics of SSR for *P. albipinna*

Statistics	Di-	Tri-	Tetra-	Penta-	Hexa-	Total
SSR number	1926231	249028	36955	3372	1836	2217422
Percentage	86.87	11.23	1.67	0.15	0.08	-

## Conclusion

In the present study, genome survey sequencing of *P. albipinna* was conducted to investigate its genomic characteristics and identify microsatellite motifs. The genome size estimated by K-mer analysis (K = 25) was 883.8 Mb, and the heterozygosity and duplication rates were 0.289 and 0.751%, respectively. The assembled genome had a total size of 819.3 Mb, with an N50 of 2867 bp and a GC content of 41.02%. A total of 2217422 SSR motifs were identified from the genome data, among which dinucleotide motifs accounted for the majority of repeat motifs (86.87%). These data will be a useful basis for novel molecular marker development as well as for further whole-genome sequencing of *P. albipinna*.

## Supplementary Material

Supplementary Table S1

## Data Availability

The *P. albipinna* genome project has been registered in NCBI under the BioProject number PRJNA697561. The whole-genome sequence has been deposited in the Sequence Read Archive (SRA) database under accession numbers: SRS13617358 and SAMN17672856.

## Abbreviations

COI: cytochrome *c* oxidase I
MaSurCA: Maryland Super-Read Celera Assembler
ND2: NADH dehydrogenase subunit 2
NGS: next-generation sequencing
SSR: simple sequence repeat
