# Supplementary material for: Genome survey and microsatellite motif identification of Pogonophryne albipinna
Source: Biosci Rep. 2021 Jul 20;41(7):BSR20210824. doi: 10.1042/BSR20210824 (PMC8292760; doi:10.1042/BSR20210824)
Supplement: Supplementary Table S1 [file BSR-2021-0824_supp.pdf]

Supplementary Table S1. Information of SSR primers of *Pogonophryne albipinna*

Primer No.	Motif	PCR product size (bp)	Forward primer		Reverse primer	
			Sequence (5′ → 3′)	Tm (℃)	Sequence (5′ → 3′)	Tm (℃)
1	(AC)15	140	TCTAGTCGAACAGCTCAGCC	59.2	AGTGGAATGAGGATGGGAGC	59.2
2	(AC)11	139	CATGTTTATTCCCACAGATGGCA	59.3	AATGTGAGGAGTTCAGGCCC	59.7
3	(AC)10	150	GGCAAGCTTTGTGTTCAGCT	59.6	ATGGACAATGAAAGGCGGGA	59.7
4	(AG)12	110	TCCCTCAACTTGTTCTTCTGGT	59.2	TCTAAGACCATGCTGACGGC	59.8
5	(AC)14	125	TCCATTCATCCTTGCCTGCT	59.4	ACAGCAGCCACATTAGACCA	59.3
6	(AC)14	142	ATTGATCAGCATTGCACCGC	59.9	CTCACTGCCAACATCAGGGT	60.0
7	(AC)10	133	GGAGCCTACAGTCCAATCGG	59.9	ACCAAGGTTTCAGAGCAGCA	59.8
8	(AC)10	135	GAGCCTACAGTCCAATCGGG	59.9	AGCACCAAGGTTTCAGAGCA	59.8
9	(AC)10	148	ACGTTTGAACCTGAAATGGGC	59.7	TGGCTCATTAGTGGTGCAGA	59.0
10	(AC)12	142	GCCTTACTTTCTGACAGCAGC	59.5	ACTGAGCTCATGTGAGGACG	59.5
11	(AC)11	150	AGTAAACTGCTGCCAAGGCT	59.9	TATCTGTCTGGCACCACCTG	59.1
12	(AC)11	135	CGTTTGATCTTGCCTGCAGG	59.8	CACCAGGTAACCTCAGCAGA	59.0
13	(AC)16	150	AGACACTCAAACTCTTAGACGCT	59.4	TCTCCGAGTGCAATGAGACG	59.8
14	(AC)10	137	CCACCGTGTTAGAAACGCAG	59.5	TTCACTGCTAAGCCAAGGCA	59.9
15	(AC)11	140	GCACCAGGATAGTCAGCACA	59.8	AGCACCTTTCACCTTGAGGG	59.9
16	(AC)11	108	ATCACCCAGGATAACCACGC	59.8	GTGGGCCTTACATCTGCTCA	59.7
17	(AG)10	118	CACGGTGGTTAAATCAGATTGGG	59.9	TAGGCCATGCAGTCATCACC	59.8
18	(AC)10	136	GCAGGATATGGGTGTTTGCG	59.6	TAGCGTCTTTGCTCAGTGCT	59.7
19	(AC)18	141	GGTATCGCTAACACCACCCA	59.5	CCGTTTGGTTAATGTCTTCGCT	59.5
20	(AC)11	140	CCAGAGCTGCTATCAGTGCA	59.8	TAGAGATGAGAGGCGGTGGT	59.7
21	(AC)15	116	GAAGCTTAGGTTTGCCTGGC	59.5	TTTAGCTCCTTCGCACCAGT	59.3
22	(AC)12	141	GCTCCATGGTGAACTTCTGC	59.2	CAGCAGAATTGAGTTAACGGCA	59.5
23	(AC)12	140	TCCATGGTGAACTTCTGCGT	59.6	ACAGCAGAATTGAGTTAACGGC	59.5
24	(AC)13	142	AGGTTTAAAGTTGTACATTTCCGCA	59.7	GGAGGACATACTGTGGTGCA	59.4
25	(AT)15	150	ATACGAGTCAGTGTGAGGCC	59.2	GGCTGGATCTCAGAAAGGTCA	59.4
26	(AC)11	106	TGGGATGGACTCAGAGCTGA	60.0	CTGATCAGCTGCTGAGACGT	59.8
27	(AC)11	118	TCACACCCTCATTTGCTCCC	60.0	CTGACAACACAGGAGCGACT	60.0
28	(AG)16	127	TCTGTGTTCGTGTTTGTGCG	59.6	TGTATAGCCCGCAAACTGCT	59.7
29	(AC)13	150	TTCTGGTCAAAGGCAACGGA	59.8	GGCTGATGGAAGATGACAGGT	59.8
30	(AC)11	123	GCTCCGTCTCTTTGAGCTCA	59.8	CAACCGGCTGATAAACACCG	59.6
31	(AC)11	142	TGTCCGTTACAACTGAGTACTGG	60.0	TCCACTCCAATGAGCTCTGC	59.7
32	(AC)10	123	CTGCAATAACAGGCCAGCAC	59.8	TTTGGAGGCACAGAGACCAC	59.9
33	(AC)11	119	GTCTCAGATACAGAAAGACAGGCT	59.8	TGTTGATTGGAGGGAGCAGG	59.7
34	(AC)10	138	ACCATGCCATGTTAGCGCTA	59.8	TGGACATTCCCACATGCACA	59.9
35	(AC)17	129	CATGGCTTCTGAAGGAGGCT	59.7	CCCTGTTAGATTGATGGGAGGG	59.9
36	(AC)11	143	CCCTTTCATCCGTTACATGCTG	59.6	TCTGAGATCTCTAGTCAGGGCA	59.5
37	(AG)16	139	TGTCTATCTGCCTTTCTGCCA	59.1	TCACCGTCATGCTAATCCTGG	59.9

38	(AT)10	141	TCTGCACATTGGTCTTGCAAC	59.7	TCCTCGTATGTGTAAACCTACCTG	59.6
39	(AC)11	122	TGGCTCTAGATCTACTCCGGG	59.9	GCATTACAGGGCATGAAGGC	59.6
40	(AC)16	138	GTTCAGAGGCAGACAGAGGG	59.8	AGGTGAACACAGACTCTGGC	59.6
41	(AC)10	139	AGCAGTGTTTACAGGCTCCC	60.0	ACCATGTCTCATCTGGACACC	59.4
42	(AC)10	140	ATCAGAAACACATTTGCCGCT	59.1	TGCAGCATTGAACACAATGGG	60.0
43	(AC)17	125	TCTGTGAGGTCGTTCAGCTG	59.7	CCTGCATGTCTGTGTTTGTCC	59.7
44	(AC)12	131	AGGCACATTTATCCACCGCT	59.7	ACAGGCCTTTCACATGCTCA	59.9
45	(AG)26	150	ACTCTGAAATGACACCGTGCT	59.9	AGCATGTGCCAATCAAACGG	59.8
46	(AC)26	143	CAGGGTACTCTCCATGGTGC	59.8	GGCAACTCTAATAGATGACTCATGC	59.6
47	(AC)11	123	CCTCAGATTGGATTCCAGAGCA	59.8	CTTGGTGTTGGAGAGGAGGG	59.7
48	(AC)12	134	GCTTCATGACCACCAAGGGA	60.0	AGGAGAGCCAATGTTAGCCG	59.8
49	(AT)11	123	CAACCAACAAGAGGCACGTC	59.7	TCGCAAGAGGAGATGATGCC	59.9
50	(AC)17	123	ACAAGGACTACTGGCCAAGC	60.0	TAATTCATCGCCATGCACGC	59.7
51	(AC)25	140	ATTTCAGCCATCCATCAGCCT	59.8	GATTCAACGCAGGTCTGACG	59.3
52	(AC)11	142	TATAAGCCCTGCAGCACGAG	59.9	AATGAGCTTTACGATGGCGC	59.3
53	(AC)14	136	GTGCATTCTGTCCTCTGTTGC	59.8	CGTGCTAATCTCCCAGAGGG	59.6
54	(AC)10	118	GTCCAGACACTGAGAGGCTG	59.8	ACGTGTAAGTGCACTCGTCT	59.3
55	(AC)11	150	CAGGCAAACATAGTCCTGCAG	59.3	ACGTGCATCTGATAAGGCCT	59.2
56	(AC)11	136	CGCCTTAATTGTTTCACGCAC	59.0	CGCCCATATCCAGTTCTGCT	59.9
57	(AC)11	128	GCATTTCTTTAGCCTAAAGCCGA	59.6	TGGATTAAGCGGATAAGGCCC	59.9
58	(AC)10	117	TCTCCTGTGGTCTTACCCGT	59.9	AGTACTTGTCCTCCAGGTGC	59.0
59	(AC)10	128	CGACAGTTACACCTACGCTCA	59.8	CAACGCACATCCTTCATGGG	59.5
60	(AC)17	125	ACACTTCACTGGTCTGGGTG	59.5	TTTCCCTGCCATGACTCGAG	59.8
61	(AT)10	147	CCTTCCTCTGTAAGCGCTGT	59.8	TTGTTGTTCTGCCCTCCTGT	59.4
62	(AC)11	147	AACGCTATTAGCACCCTGGG	59.8	CCCTGTGAGGAGACTTCAGC	59.8
63	(AT)10	137	TCTCCATCCATCCTAAATCAGCA	59.0	AGCAAGGTCCAATAAAGAAACCG	59.5
64	(AC)11	140	ACATCTGCTTGTGTAGGCGT	59.7	CTCCACAATCCATCATGCGC	59.7
65	(AC)11	144	GGCCGCCTCTTAGTATGACC	60.0	AAGCAGAAAGAGACAAGCCCA	59.9
66	(AC)10	141	GGAGACTGAGTTTGAATCGGGA	59.8	CCCGATCTGAACAAAGATTGCC	59.9
67	(AT)11	140	TGTGATGCTAGAGTGACTCGTG	59.8	CATTGTTTACACCCGTATGAGCT	59.1
68	(AT)11	140	GTGATGCTAGAGTGACTCGTGA	59.6	ACATTGTTTACACCCGTATGAGC	59.3
69	(AC)14	140	CAATAACCTACAGCAGTGACCTG	59.1	TGCATCAGGATCTTTAACGCAC	59.3
70	(AC)14	140	AATAACCTACAGCAGTGACCTGT	59.4	CTGCATCAGGATCTTTAACGCA	59.1
71	(AC)10	141	TGCTACGGCAACTAACACCT	59.3	GAACATTCCGTACAACCAGGC	59.5
72	(AC)14	142	GTCCCAGAAAGAGCTCTGCA	59.7	TCACATTAGGAGCTACTGTAACCC	59.6
73	(AC)21	136	AGAAGGGAGCAAGGTTGAGC	60.0	GTCCAAAGAGGCTGTCGCTA	59.8
74	(AC)22	142	GGCTGTCATTCCCTTTCTGC	59.2	GCTGATCGGAGAACACAGGT	59.8
75	(AC)10	148	AAATCTCCTCCACGTGGACC	59.4	TTTGGGCTGGACCTAACTGG	59.6
76	(AC)16	142	ACACAGAGGGACAAACGTCA	59.2	ATGAGTTACGTCACCAGCCC	59.8
77	(AGC)7	144	TAGCAGAACGGTTAGCTCGG	59.5	GGGTAATGGTGACTCTGCCA	59.4
78	(AGG)8	142	ATCGATCGACAGGTCAAGGC	59.9	GGGTACTCCGCTCTAGTTGC	59.9

79	(AAT)8	144	CCACCAACATCTGTCTCCGT	59.7	TTCACCATGTAAAGCGGCCT	60.0
80	(AAT)8	144	ACCACCAACATCTGTCTCCG	59.7	TCACCATGTAAAGCGGCCTT	60.0
81	(ATC)7	138	AGCCCAATCTGAAACAGGAGG	60.0	CGTGGCTGATGTTCTTGCTG	59.8
82	(AGG)7	121	CACAGTTGACAAGGCACAGC	60.0	AGAGGAACAGGATAGGATGGGA	59.5
83	(AAT)12	139	TGTAAAGCGACCTTGGGTGA	59.2	TCGGAGTGTAGTGAGTCACCT	59.9
84	(AGG)12	141	ACCCTCTTGTCACTGACAGC	59.6	AGCTACAACTGCTGTTAGGGT	59.0
85	(AGG)12	140	CCCTCTTGTCACTGACAGCA	59.6	AGCTACAACTGCTGTTAGGGTT	59.6
86	(AAC)8	118	CTCCAAAGCCTTGTGAAGCG	59.8	TCATTGTCAGTGCTGGTCCC	60.0
87	(AGC)7	133	ACAAGATGAAGAGGCAGGCT	59.0	TTCCCATCCTTCACCAGCAC	60.0
88	(AAC)7	121	ACATGATCTCTGCAGCTGCT	59.5	AGGTAATGACACCATGCAGCA	60.0
89	(ACC)9	136	ATCCACAGACTGATCCAGCG	59.5	TCTGACATCACCTCTGCCAG	59.1
90	(AAC)8	138	TCTCCAGTCAGCTCAACACG	59.7	GAGGGATGCTCTGACTTGCA	59.7
91	(AAC)8	131	ATGTGAAGACCCTGACCTGC	59.7	ATTGTGGTGGGTGAGACAGG	59.6
92	(AAG)10	140	TTTGAAGCTGCTAACACGCAA	59.3	AGCAGTAACGTTAGAGGGCAG	59.8
93	(AGG)8	133	TCAATGCCTTATTTAACCAATGGGA	59.0	TCTGAAAGGCATCTTTGAGGCT	60.0
94	(AGG)7	143	TCCTTGTGAATGACAGCGCT	60.0	TGCATTGTTATGTTATGGTGCCA	59.2
95	(AGC)8	141	GTCACACTCAGACAGACGCT	59.7	GTGTTCTCACCTGTCTGAGCA	59.9
96	(AGG)7	140	CCTACAGTCATGGCTCAGCA	59.5	TGGAGACAGTAAAGCAGCAGG	60.0
97	(AAT)7	141	GTTTCCTAGGCCAGTCAGCT	59.4	AATGCGACCTTGAGAGCCTT	59.7
98	(AGG)9	144	GAGGTAGCAAGAGGATGGAGG	59.3	TAACTGACCTGCCACTCACC	59.3
99	(AGC)10	138	AGAGCCAAGACTAGCAGTGC	59.8	TGACGGATATCAGCTGTGGC	59.9
